# Concentrated Organic Medicines

**Published:** 1860-05

**Authors:** V. L. Hurlbut

**Affiliations:** Chicago


### "CONCENTRATED ORGANIC MEDICINES."

By V. L. HURLBUT, M. D., Chicago.

The object of this article, is to invite the attention of the members of our profession to the "*Concentrated Organic Medicines*," as prepared by B. Keith & Co. I have used these remedies to some considerable extent in my practice—as have others—and they have proven, in every instance, all that is claimed for them. They are *positive in therapeutic power*, *uniform in strength*, *prompt in action*, *and always reliable*. This cannot be said of the Crude articles, nor of the "Fl. Exts," which I presume all have used more or less, and usually with unsatisfactory results, for very obvious reasons, which every intelligent physician understands.

Although not condemning the use of mercury in toto, ourselves, yet, we all know that there is a deep-rooted prejudice against it, which we have to combat almost every day, and if there is a substitute which will accomplish all that the mineral can, without leaving any of its evil consequences, it would seem to be wise and humane to avail ourselves of it. Furthermore, the plants, flowers, and shrubs, from which these medicines are derived, are indigenous to our soil and climate, which pointedly indicates that nature designated their use in the diseases incident to our climate. We should not disregard the simplest teachings of the ancient mother, for her wonderous arcana has furnished the materials from which science has elaborated the manifold remedies for the legion of fleshy ills which are our heritage. It has doubtless been the indiscriminate and excessive exhibition of mercury, which has caused so many persons to seek a different method of treatment for themselves and families, and surely the profession should keep pace with the people; for of all classes of men, the medical should be characterized by the most enlarged liberality; for no other assumes such fearful responsibilities; they constitute themselves *conservators of health*, consequently of *life*. We that have assumed this position, should better understand and appreciate the near compatibility of "*Organic Medicines*," with the functions of organic life, and not be so wedded to an *ism* as to be blinded to all light and truth that does not descend in the direct line from Hippocrates. As a profession, we are too apt to be severe in our judgments upon any and everything that may chance to have originated in our own country, and particularly so, if introduced by a sect other than our own; but let the same thing emenate from the "*East*," bearing the prestige of antiquity, and we are very willing to give it at least a fair trial before condemning it.

I do not propose to discuss the modus operandi of the "organic remedies," but honestly wish that all our members would give them a thorough and impartial investigation. In expressing this wish, I am prompted by no feeling other than earnest desire—in which I believe you all sympathize—to promote the best interests of our common humanity.

